# A Defined Terminal Region of the *E. coli* Chromosome Shows Late Segregation and High FtsK Activity

**DOI:** 10.1371/journal.pone.0022164

**Published:** 2011-07-20

**Authors:** Marie Deghorain, Carine Pagès, Jean-Christophe Meile, Mathieu Stouf, Hervé Capiaux, Romain Mercier, Christian Lesterlin, Bernard Hallet, François Cornet

**Affiliations:** 1 Laboratoire de Microbiologie et de Génétique Moléculaire, CNRS, Toulouse, France; 2 Université de Toulouse, Université Paul Sabatier, Toulouse, France; 3 Université Catholique de Louvain, Institut des Sciences de la Vie, Unité de Génétique, Louvain-La-Neuve, Belgium; University of Massachusetts Medical School, United States of America

## Abstract

**Background:**

The FtsK DNA-translocase controls the last steps of chromosome segregation in *E. coli*. It translocates sister chromosomes using the KOPS DNA motifs to orient its activity, and controls the resolution of dimeric forms of sister chromosomes by XerCD-mediated recombination at the *dif* site and their decatenation by TopoIV.

**Methodology:**

We have used XerCD/*dif* recombination as a genetic trap to probe the interaction of FtsK with loci located in different regions of the chromosome. This assay revealed that the activity of FtsK is restricted to a ∼400 kb terminal region of the chromosome around the natural position of the *dif* site. Preferential interaction with this region required the tethering of FtsK to the division septum via its N-terminal domain as well as its translocation activity. However, the KOPS-recognition activity of FtsK was not required. Displacement of replication termination outside the FtsK high activity region had no effect on FtsK activity and deletion of a part of this region was not compensated by its extension to neighbouring regions. By observing the fate of fluorescent-tagged loci of the *ter* region, we found that segregation of the FtsK high activity region is delayed compared to that of its adjacent regions.

**Significance:**

Our results show that a restricted terminal region of the chromosome is specifically dedicated to the last steps of chromosome segregation and to their coupling with cell division by FtsK.

## Introduction

Bacterial chromosomes consist of single replication units and are organised in two replichores of opposite polarity from the replication origin (*ori*) to the opposite termination region (*ter*), which has a profound impact on their dynamics [1]. Segregation of chromosome loci occurs shortly after their replication and thus occurs progressively along the replichores [2], [3]. The *ori* and *ter* regions are the sites of specific activities dedicated to the initial and final steps of segregation, respectively. In *E. coli*, the final steps of segregation occur shortly before and during cell division. They involve the removal of catenation links by TopoIV topoisomerase and resolution of chromosome dimers by the XerCD/*dif* site-specific recombination system [4], [5]. The FtsK protein, a DNA translocase associated with the division septum, controls both activities. FtsK, TopoIV and the Xer recombination system are highly conserved in bacteria and have been shown to play roles similar to their *E. coli* homologs in several evolutionary remote organisms [6], [7], [8], [9], [10].

FtsK is an ATP-driven dsDNA-translocase required for both cell division and faithful chromosome segregation (reviewed in [11], [12]). In *E. coli*, different domains of FtsK achieve its different activities. Its N-terminal domain, FtsK_N_, is essential for growth and cell division, and contains transmembrane helices that link FtsK to the division septum [13], [14]. The central domain, FtsK_L_, is non-essential for growth but is involved in interactions with other cell division proteins [15], [16], [17], [18]. The C-terminal domain, FtsK_C_, is also dispensable but involved in chromosome segregation. It carries Walker-type ATPase motifs and forms a hexameric motor that translocates dsDNA [19], [20]. It also interacts with TopoIV and activates its decatenation activity *in vitro* [21], [22]. FtsK_C_ can be subdivided into three subdomains based on their structure, function and sequence conservation [20], [23]. The FtsKα and FtsKβ subdomains form the translocation motor while the extreme C-terminal subdomain, FtsKγ, controls the translocation activity [24]. FtsKγ contains a winged-helix DNA-binding domain that recognises specific DNA motifs, the KOPS [23], [25], [26], [27]. KOPS motifs are inversely oriented on the two chromosome replichores, thereby directing translocation towards the *dif* dimer resolution site located at the terminal replichore junction [26], [28], [29], [30], [31]. FtsKγ also interacts with XerD and activates XerCD/*dif* recombination [32], [33], [34].

The FtsK_C_ motor assembles as a hexamer upon interaction with DNA [27]. Although it can interact with non-specific DNA, FtsK_C_ preferentially interacts with the KOPS motif, which orients translocation at the loading step [26], [27], [30]. The fact that KOPS motifs are over-represented and their orientation biased towards the *dif* site along the entire chromosome [28] raises the question of the interaction of FtsK with the different chromosome regions. The *ter* region is susceptible to high frequencies of DNA breakage, which are thought to occur specifically in unresolved dimers [5]. Interestingly, the region concerned by DNA breakage is larger in an *ftsK_C_* mutant than in a *xer*- mutant [35]. From these data, it was postulated that FtsK is involved in the positioning of a 250 kb region extending anti-clockwise from *dif* near the septum (analyses was not extended to the clockwise side). We have used XerCD/*dif* recombination to probe the interaction of different chromosome loci with FtsK. This revealed that FtsK acts in a specific ∼400 kb region around the natural position of *dif*, thereby defining a region of preferential FtsK activity. The preferential interaction of FtsK with this region depends on both the translocation activity of FtsK and its tethering to the division septum, but not on its KOPS-recognition activity. Although replication normally terminates close to *dif*, displacement of termination did not modify the activity of FtsK in this region. Deletion of parts of the FtsK high activity region or its adjacent regions led to its shortening rather than its extension to adjacent regions. We also show that the segregation of sister FtsK high activity region is delayed compared with that of adjacent regions. We conclude that a restricted region of the chromosome is specifically dedicated to the last steps of segregation and to its coupling with cell division by the FtsK protein.

## Results

### FtsK acts in a restricted region of the chromosome

**Figure 1:**
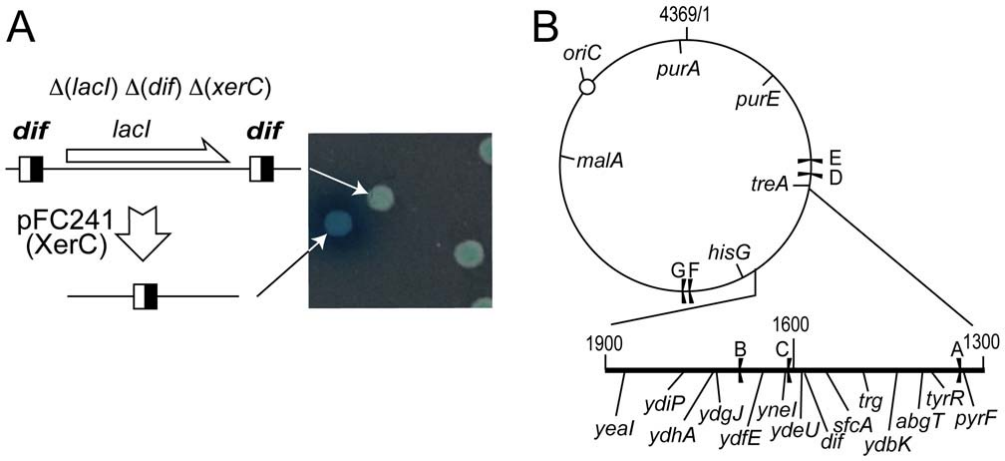
Measuring FtsK Activity. (A) The *dif-lacI dif* cassette is shown with the *dif* sites as black and white squares. It was inserted at chosen loci of a Δ(*xerC*)Δ(*dif*)Δ(*lacI*) strain. Transformation with the XerC-producing plasmid pFC241 allows recombination, giving rise to blue colonies on indicator medium (right). (B) Map of the chromosome with the insertion loci used for insertion of the *dif-lacI dif* or *parS*-Kn cassettes. The replication origin (closed circle) and replication terminators (black flags) are indicated. Coordinates are in Kb.

Recombination between *dif* sites requires a direct interaction between FtsKγ and XerD [32], [33]. We reasoned that XerCD/*dif* recombination could be used to measure the relative frequencies at which FtsK interacts with different chromosome loci. To this end, we constructed a *dif*-*lacI*-*dif* cassette and inserted it in the chromosome of a strain deleted for *dif*, *lacI* and *xerC* (Figure 1A; Materials and methods). Recombination between *dif* sites was induced by transformation with the XerC-producing plasmid pFC241, which provoked the loss of *lacI* and derepression of the *lacZ* gene thus allowing the measurement of recombination frequencies from the formation of blue colonies on indicator plates (Figure 1A).

**Figure 2:**
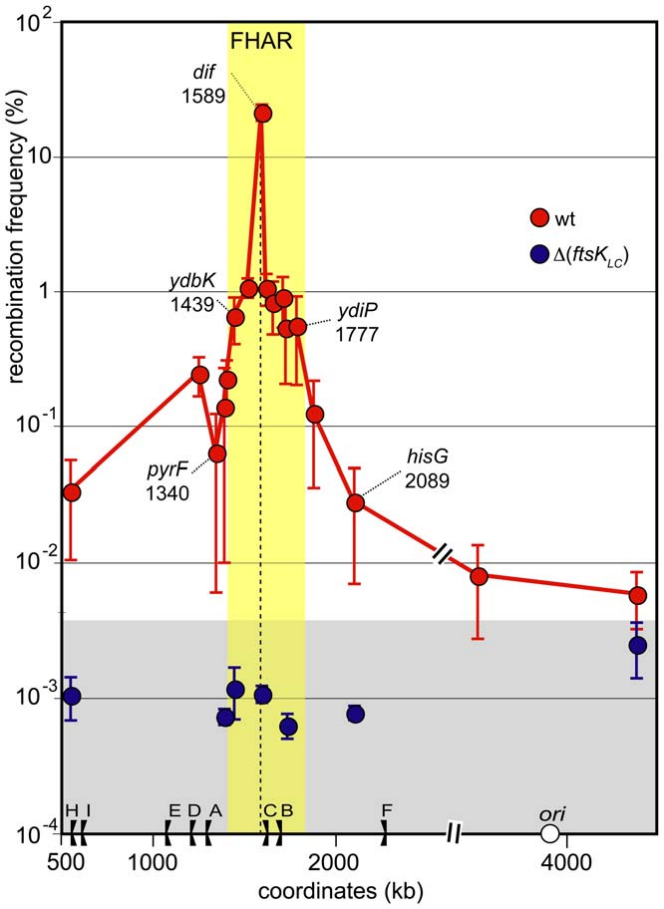
FtsK acts in a restricted region of the chromosome. The *dif-lacI-dif* cassette was inserted at chosen loci and recombination was scored. Y-axis: recombination frequency measured at each locus (calculated frequency of recombination per generation; mean of 5 independent measurements with standard deviation; Materials and methods). X-axis: coordinate of the insertion loci (positions of a subset of loci are shown on the graph). The dashed line indicates the natural position of *dif*, the flags the replication terminators with corresponding letters (A to F) and the open circle the replication origin. Coordinates are in kb. Red dots: *ftsKwt* strains; blue dots: Δ(*ftsK*_LC_) strains. The yellow zone indicates the extent of the FtsK high activity region and the grey zone the FtsK-independent recombination background.

The *dif*-*lacI*-*dif* cassette was inserted at 18 different chromosome loci (Figure 1B; Materials and methods). Recombination was scored in the resulting strains and in Δ(*ftsK_LC_*) derivatives to measure the level of FtsK_C_-dependent recombination. As previously reported, FtsK_C_-dependent recombination was most frequent at the natural position of *dif* (Figure 2). This locus is the only one assayed inside the previously defined *dif* activity zone (DAZ), which is the zone where oppositely oriented KOPS converge, where inserted *dif* sites can resolve chromosome dimers efficiently [28], [36], [37]. FtsK reaches this locus at least in every cell harbouring a chromosome dimer (i.e. about 15% of the cells/generation in these growth conditions [37]), hence the high frequency of recombination observed. Recombination was low (<0.1%) at loci located more than 200 kb from the natural *dif* position (Figure 2). Nevertheless, comparison with Δ *ftsK_LC_*) strains showed that these low levels of recombination still largely depend on FtsK_C_ (compare blue to red dots in Figure 2). FtsK can thus interact with most of the chromosome, although very infrequently with loci far from the natural *dif* position. Interestingly, loci located within a ∼400 kb long region around the natural position of *dif* displayed higher frequencies of FtsK_C_-dependent recombination (about 1%; Figure 2). In this region, the recombination frequencies only slightly decreased with increasing distance from the natural *dif* position whereas frequencies decreased abruptly in the adjacent regions. Notably, *dif* sites inserted at all positions but the natural *dif* position do not resolve dimers efficiently [37], implying that most recombination events occurred on monomeric chromosomes. We conclude that FtsK preferentially interacts with a ∼400 kb long located in the terminal part of the chromosome.

### Roles of the different FtsK domains

**Figure 3:**
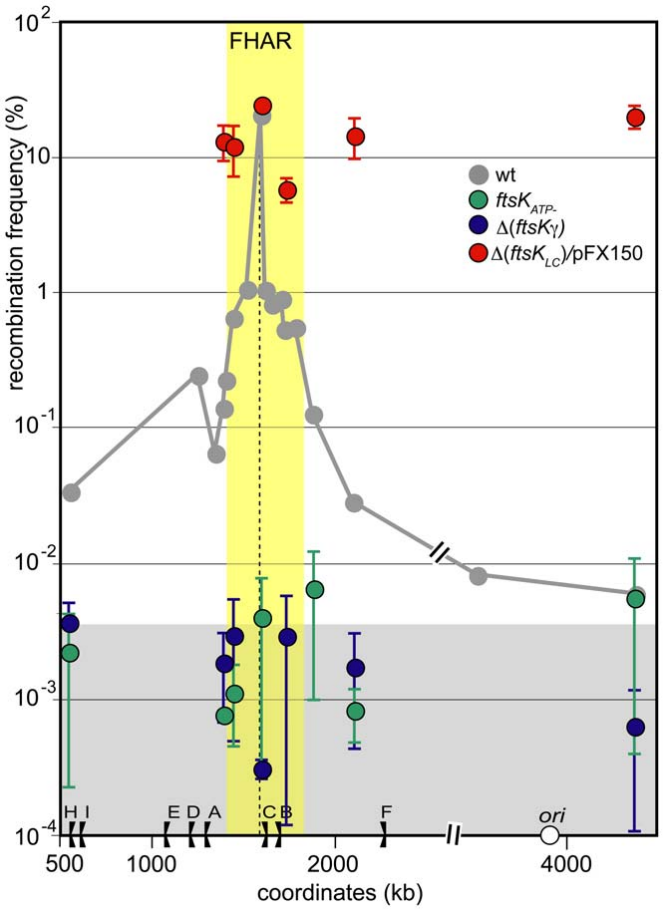
Preferential interaction with the FtsK high activity region requires all domains of FtsK. Same legend as Figure 2. The grey curve corresponds to *ftsKwt* strains and is redrawn from figure 2. Recombination frequencies were measured at chosen loci in different *ftsK* mutant: Green dots: *ftsK*_ATP-_; blue dots: Δ(*ftsKγ*); red dots Δ(*ftsK*_LC_) strains producing FtsK_C_ from plasmid pFX150 (Materials and methods). The yellow zone indicates extend of the FtsK high activity region and the grey zone the FtsK-independent recombination background (see Figure 2).

To investigate which features of FtsK are required for its preferential interaction with the FtsK high activity region, we first produced FtsK_C_ detached from FtsK_N_ from plasmid pFX150 in Δ(*ftsK_LC_*) strains (Materials and Methods). Production of FtsK_C_ was induced using 0.03% arabinose, which yields a low but detectable level of protein [19]. As expected, this resulted in high recombination frequencies (red dots in Figure 3). Importantly, recombination frequencies were equivalent at all loci assayed (Figure 3). These results show that FtsK_C_ can interact with the different chromosome regions at equivalent frequencies when unlinked from the division septum. It also follows that the XerCD recombinases have equal access to *dif* sites inserted into the different chromosome regions. We infer from these data that the restriction of FtsK activity to the FtsK high activity region depends on the tethering of FtsK to the division septum by FtsK_N_.

FtsK_C_ exhibit three distinct activities: it translocates DNA via the FtsKαβ motor, it recognises KOPS and induces XerCD/*dif* recombination via the FtsKγ subdomain. We used *ftsK* mutant alleles to separate these activities. The Δ(*ftsKγ*) allele encodes a protein that translocates DNA but neither recognises KOPS nor induces recombination [27], [38]. Consistently, recombination frequencies in Δ(*ftsKγ*) strains were as low as in Δ(*ftsK_LC_*) strains at all loci assayed (blue dots in Figure 3), confirming that no FtsK-dependent induction of XerCD/*dif* recombination can occur in the absence of FtsKγ. The *ftsK_ATP_*_-_ allele encodes a protein that does not translocate DNA but carries an intact FtsKγ subdomain. Recombination frequencies in *ftsK_ATP-_* strains were undistinguishable from those in Δ(*ftsK_LC_*) and Δ(*ftsKγ*) strains (green dots in Figure 3). We conclude that recombination induction requires both an intact FtsKγ subdomain and DNA translocation activity at all loci assayed. It follows that our recombination assay measures the capacity of FtsK to translocate the different regions of the chromosome rather than just to interact with the XerCD/*dif* complexes they carry.

### Role of the KOPS recognition activity of FtsK

**Figure 4:**
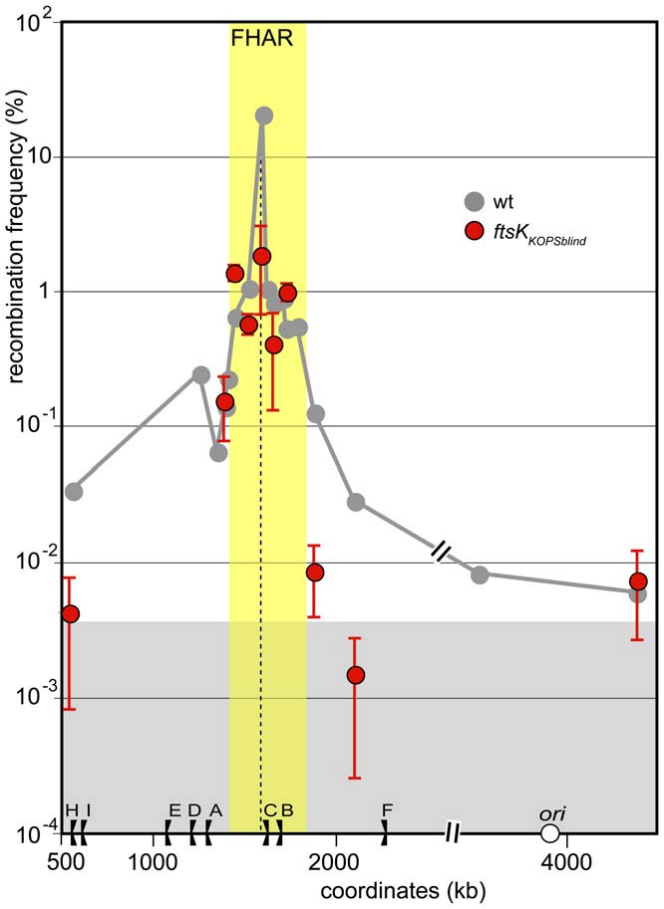
KOPS recognition is not required for preferential interaction of FtsK with the FtsK high activity region. Same legend as Figure 2 and 3. Grey curve: *ftsK*wt, redrawn from figure 2; red dots *ftsK*_KOPSblind_. The yellow zone indicates the FtsK high activity region and the grey zone the FtsK-independent recombination background (see Figure 2).

Although FtsK can recognise and translocate any DNA *in vitro*, it does so preferentially with KOPS motifs [23], [27], [30]. This prompted us to investigate how the KOPS recognition activity controls the interaction of FtsK with the chromosome. We used the *ftsK_KOPSblind_* allele of FtsK, which does not recognise the KOPS motif and so translocates DNA in a non-oriented manner [23], [31], [33]. Inactivating KOPS recognition lowered recombination at the natural *dif* position (red dots in Figure 4). This is consistent with a reduced capacity of the FtsK_KOPSblind_ protein to reach the position where KOPS orientations converge compared to the wild type FtsK and with the accompanying partial defect in chromosome dimer resolution in *ftsK_KOPSblind_* strains [31]. In contrast, recombination frequencies appeared unchanged at loci located inside the FtsK high activity region or in its left transition zone (compare red to grey dots in Figure 4). This shows that KOPS recognition is not required for FtsK to translocate this region. Importantly, the FtsK high activity region did not expand in *ftsK_KOPSblind_* compared to wt strains, showing that the restriction of FtsK activity to this region is not due to KOPS-mediated orientation of FtsK translocation. Indeed, recombination frequencies at loci located outside the FtsK high activity region were undistinguishable from that of FtsK-independent recombination (Figure 4). Thus, the FtsK_KOPSblind_ protein is unable to translocate DNA at loci located outside the FtsK high activity region whereas the wild type FtsK can do so, although at a low frequency.

### No role for termination of replication

**Figure 5:**
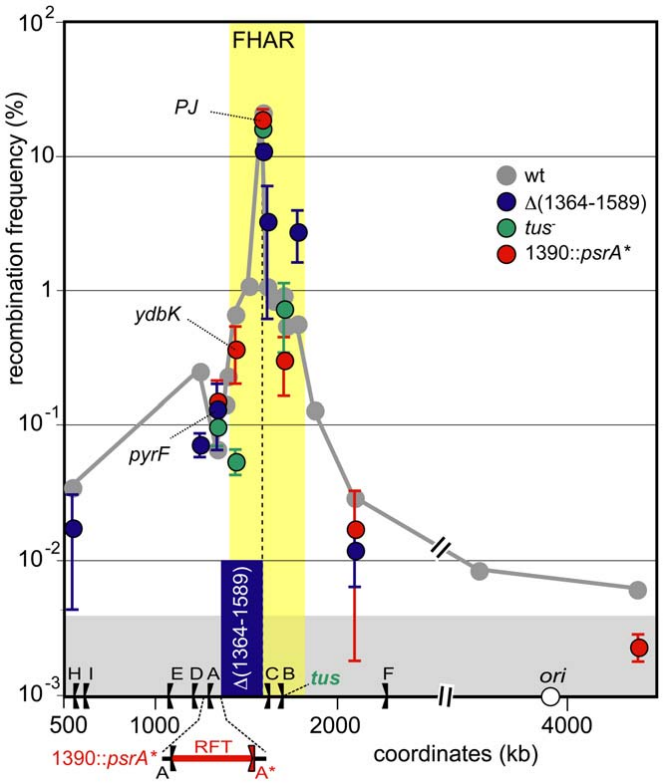
Specificity of the FtsK high activity region. Same legend as Figure 2 and 3. Green dots: strains were mutated for the *tus* gene. The position of the *tus* gene is indicated on the X-axis. Red dots: An additional replication terminator, psrA* (A*), was inserted at position 1390. The resulting restricted replication fork trap (RFT) is shown in red bellow the X-axis. Blue dots: strains were deleted of the 1364–1589 fragment (Materials and methods). The deleted fragment is shown as the blue bar. Positions of a subset of loci are shown on the graph. PJ indicates the junction of KOPS polarity (i.e., the endpoints of the deletion in the Δ(1364–1589) strain and the natural position of *dif* in the other strains. The yellow zone indicates extend of the FtsK high activity region and the grey zone the FtsK-independent recombination background (see Figure 2).

The FtsK high activity region is the last chromosome region replicated. Half of it is contained in the replication fork trap (the zone between TerA and TerC, Figure 2). In most cells, replication terminates in the vicinity of the TerC terminator, which is located near the natural *dif* position [39], [40]. We examined a possible role for termination of replication in the control of FtsK activity by inserting an extra replication terminator, psrA*, at position 1390 kb (Materials and methods). This restricts termination to a 50 kb zone located outside the FtsK high activity region (Figure 5). Insertion of psrA* affected only slightly if at all the recombination frequencies at the loci assayed (red dots in Figure 5). In particular, the *ydbK* locus is located inside the replication fork trap in wt strains and outside it in psrA* strains whereas the *pyrF* locus is far from most termination events in wt strains and close to them in strains harbouring psrA* (Figure 5). Insertion of psrA* did not significantly change the recombination frequencies at these two loci. Moreover, inactivation of the Tus protein, which is required for termination at terminator sites, had no significant effect on recombination frequencies (green dots in Figure 5). We conclude that the high activity of FtsK does not result from the location of replication termination in the concerned region but rather is linked to its post-replicative processing.

### The FtsK high activity region can be shortened by deletion

A large terminal part of the chromosome, from 1349 to 1712 kb, contains no essential gene and can be deleted [41], [42]. This prompted us to observe the effect of deleting a part of the FtsK high activity region. We constructed a large deletion of 224 kb to the left of the natural *dif* position that contains the left part of the FtsK high activity region and its transition zone (Materials and Methods; Figure 5). Recombination frequencies were then scored at different loci in strains carrying this deletion. As expected, the endpoints of the deletion supported a high frequency of recombination because it is a new zone of converging KOPS (polarity junction (PJ) in Figure 5; [37]). However, this frequency were slightly less than in the wt strain at the *dif* position, suggesting that deletion of half of the FtsK high activity region lowers the capacity of FtsK to reach the zone of converging KOPS. Microscopic observation did not reveal filament formation in the deletion strain carrying the *dif* site in the zone of converging KOPS, indicating that resolution of chromosome dimers was not affected by the deletion (data not shown). Recombination frequencies at loci located inside the remaining part of the FtsK high activity region were higher than in the absence of deletion (blue dots in Figure 5). Thus, FtsK appears to translocate loci of the shortened FtsK high activity region more often than the same loci in the wt strain. Recombination frequencies at loci located outside the FtsK high activity region did not differ significantly from those of the wt strains (Figure 5). Notably, this is the case at the *pyrF* locus, which is located only 24 kb away from the zone of converging KOPS in the strain carrying the deletion. We conclude that deletion of the left half of the FtsK high activity region does not provoke its extension to adjacent sequences but rather its shortening.

### The FtsK high activity region displays a specific segregation pattern

**Figure 6:**
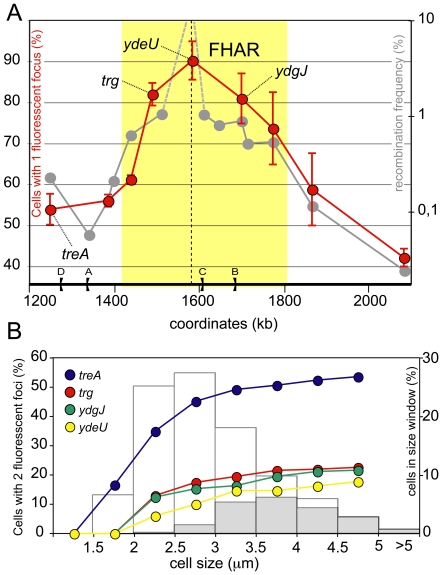
Delayed segregation of the FtsK high activity region. (A) Strains carrying a *parS* insertion at chosen loci and producing the GFP-Δ30ParB protein were observed under the microscope and the ratio of cells with 1 fluorescent focus on total cells was calculated (reft dots; left Y-axis; Materials and methods). The means of at least 3 independent measures with standard deviation are plotted (n>250 cells for each measure). The grey curve and yellow zone are redrawn from Figure 2 for comparison, omitting the *dif* natural position (right Y-axis). The dotted line indicates the natural *dif* position. (B) Data from (A) were sampled in cell size windows (0.5 µm each, X-axis) and the fraction of cells with 2 segregated fluorescent foci was plotted (left Y-Axis) for the four representative loci indicated. The distributions of cells in the same cell size windows is shown as histograms (right Y-axis). Open bars: total cells; Grey bars: cells harbouring a constricting division septum.

The terminal region of the chromosome is the last to be replicated and segregated. Numerous studies have shown that loci of the terminal region tend to remain at the cell centre long after their replication before being segregated to daughter cells [43], [44], [45], [46]. This prompted us to observe the segregation timing of loci located within or outside the FtsK high activity region. Loci were tagged with the bacteriophage P1 *parS* site, which allows them to be detected by microscopy as fluorescent foci upon production of the GFP-Δ30ParB fluorescent protein that specifically binds *parS* [47]. Strains harbouring *parS* insertions were grown to exponential phase in conditions reported to avoid the intrinsic cohesion due to the *parS*/GFP-Δ30ParB system and examined by fluorescence microscopy [48] (Figure S1; Materials and methods). In these conditions, neither the insertion of the *parS* site nor the production of the GFP-Δ30ParB protein had detectable effects on the doubling time or on the shape and length distribution of cells of any of the strains used (data not shown). For better comparison with the recombination frequencies pattern, we measured recombination at the different loci in the growth conditions used for microscopy. This did not significantly modify the recombination frequency at any locus (data not shown). As all loci assayed where near the replication terminus, the vast majority of the cells harboured one or two fluorescent foci (fewer than 3% of the cells had more than two foci; Figure S1). For each strain, we measured the fraction of cells harbouring a single fluorescent focus, which increases directly with the time taken by the different loci to segregate. The results presented in figure 6A show that the fraction of single-focus cells was higher for loci located inside the FtsK high activity region than for loci outside this region. To corroborate this observation, we analysed the segregation of fluorescent foci as a function of cell size and of the presence of division septa, both indicators of progression of the cell cycle. Results are presented figure 6B for four representative loci, the *ydeQ*, *trg* and *ydgJ* loci located inside the FtsK high activity region and the *treA* locus located outside this region. As expected, the *ydeU*, *trg* and *ydgJ* loci showed equivalent segregation patterns and segregated in cells about to divide or dividing. In contrast, the *treA* locus displayed a clearly different pattern and segregated earlier, mostly in non-dividing cells.

## Discussion

The results reported here show that a specific region of the *E. coli* chromosome is dedicated to the last steps of chromosome segregation and to their coupling to cell division via the action of the FtsK protein. This region is subjected to higher frequencies of processing by the division septum-associated FtsK DNA translocase than the rest of the chromosome. Consistent with this, segregation of this FtsK high activity region is delayed compared with that of its adjacent regions. This indicates that it stays longer in the midcell zone where the growing division septum allows activation of FtsK_C_ activities. Notably, the preferential action of FtsK this region depends neither on where replication terminates nor on the KOPS-reading activity of FtsK. These results have implications for both the way FtsK interacts with and processes the *E. coli* chromosome and the segregation of the *ter* region.

### Interaction of FtsK with the chromosome

We assayed the interaction of FtsK with the chromosome by scoring recombination between two *dif* sites inserted in direct repeat at chosen loci. This assay measures a physical interaction between FtsK and the assayed locus since XerCD/*dif* recombination requires direct interaction between XerD and the FtsKγ subdomain [24], [32]. In addition, we recently reported that FtsKγ can induce XerCD/*dif* recombination when produced in the absence of the FtsKαβ translocation motor [33], raising the possibility that our assay measures FtsKγ-XerD interactions in the absence of translocation. We however ruled out this possibility by showing that the FtsK_ATP-_ protein, which contains an intact FtsKγ subdomain but is unable to translocate DNA, did not induce recombination in any region of the chromosome. Consistent with this observation, purified FtsK_C_ activates XerCD/*dif* recombination in vitro only in the presence of ATP and if one of the *dif*-carrying DNA substrates is long enough for FtsK to bind to it and translocate [28], [30], [49], [50]. It follows that although FtsKγ alone can induce recombination, its activity is restricted to translocating FtsK hexamers when linked to the FtsKαβ motor. We conclude that our assay measures the ability of FtsK to translocate DNA at the different loci assayed.

FtsK is associated with the cell division septum via its N-terminal domain, FtsK_N_ [Yu, 1998; Wang, 1998]. Although required for the resolution of chromosome dimers [33], [51], this tethering is not necessary for induction of XerCD/*dif* recombination since production of FtsK_C_ detached from FtsK_N_ induces XerCD/*dif* recombination [52]. In contrast to wt FtsK, FtsK_C_ alone acts with similar efficiency in the different regions of the chromosome. This shows that the tethering of FtsK to the division septum acts to restrict FtsK activity to the terminal region. It follows that in wild type cells, most active FtsK is septum-associated.

The FtsK_C_ motor binds to any DNA in vitro but does so preferentially onto KOPS motifs [26], [27], [30]. The KOPS-recognition activity of FtsK is, however, not required for its preferential interaction with the FtsK high activity region since the FtsK_KOPSblind_ protein appears to translocate loci inside this region with the same efficiency as wt FtsK. Consistent with this observation, KOPS are over-represented and their orientation is biased all around the chromosome, and the density of KOPS is similar inside and outside the FtsK high activity region (one 5'-GGGNAGGG motif every ∼12 kb). In addition, FtsK_C_, which carries the KOPS recognition domain, has equal access to loci located inside and outside the FtsK high activity region when not tethered to the division septum. The preferential action of FtsK thus does not imply the FtsK-KOPS interaction. The position of a locus with respect to the terminal junction of KOPS polarity (i.e., the *dif* position in wt strains) is also unimportant for the activity of FtsK at this locus. This can be inferred from the fact that the low recombination frequencies observed at loci normally far from the KOPS junction in wt strains remain unchanged when these loci are close to this junction after deletion. These data also suggest that multiple internal elements rather than unique borders determine the FtsK high activity region.

Although the FtsK_KOPSblind_ protein shows the same activity as wt FtsK in the FtsK high activity region, its capacity to reach the *dif* natural position is affected, as indicated by the lower recombination frequency in the *ftsK_KOPSblind_* mutant compared to wt at the *dif* locus and by the partial deficiency in chromosome dimer resolution of this mutant [31]. This indicates that KOPS are not required for the preferential loading of FtsK in the FtsK high activity region but only for the orientation of subsequent translocation towards the *dif* position. Lastly, FtsK translocates loci located outside the FtsK high activity region at low but significant frequencies. This activity may be due either to rare active FtsK hexamers unlinked to the division septum or to a sporadic presence near the division septum of loci located outside the FtsK high activity region. In both cases, this may be linked to a role for FtsK independent of its roles in the last steps of segregation, for instance in replication or in the repair of broken chromosomes. Whatever this role, it depends strongly on the KOPS-recognition activity of FtsK since recombination frequencies at loci outside the FtsK high activity region requires this activity. This may explain why KOPS are over-represented and their orientation biased in all chromosome regions.

### Segregation of the *ter* region

FtsK is recruited early to the division septum, at a time roughly corresponding to termination of replication [14], [53]. It is however unable to induce XerCD/*dif* recombination at this time. FtsK-dependent recombination occurs later and appears to depend on constriction of the division septum [54], [55]. Our results imply that, at the time of division, loci located inside the FtsK high activity region are more often located in the direct vicinity of the division septum than other loci. This is consistent with current models for segregation of the *E. coli* chromosome in which the *ter* loci are replicated in the mid-cell region and tends to keep this central position until septum constriction [43], [44], [45], [46], [53], [56], [57]. Using direct visualisation of the sub-cellular localisation of different *ter* loci, we have shown that this phenomenon is enhanced for loci located inside the FtsK high activity region compared to loci of its neighbouring regions. Indeed, the fraction of cells harbouring a single fluorescent focus of a tagged locus is enhanced inside the FtsK high activity region. Importantly, these different segregation patterns are not attributable to the replication timing of loci. For instance, two loci located 100 kb apart are predicted to be replicated within less than two min. by a single replication fork, which would alter the fraction of single-focus cells less than 2% in the growth conditions we used. Alternatively, the delayed segregation of the FtsK high activity region might be due to delayed or slower replication of this chromosome region. However, studies of the replication timing of *ter* loci militate against a significantly delayed replication of loci inside this region (for instance, the TerC locus [58]). The increase of the single-focus cell fraction for loci inside the FtsK high activity region is thus not due to delayed replication but rather to a slower post-replicative segregation of this region compared to its neighbouring regions. Consistent with this conclusion, a displacement of replication termination outside the FtsK high activity region does not modify its preferential processing by FtsK. Delayed segregation implies that the FtsK high activity region stays longer in the vicinity of the division septum than other chromosome regions. This proximity creates a preferential substrate for FtsK, which thus interacts efficiently with this region even when devoid of its KOPS-recognition activity. On the other hand, the FtsK high activity region may exclude other chromosome regions from the septum vicinity, which would explain their rare interaction with FtsK and its strict requirement for the KOPS-recognition activity.

What is the basis of the delayed segregation of the FtsK high activity region? Since FtsK is attached to the septum, its interaction with this region may result in delayed segregation. In this view, FtsK would interact with sister chromosome irrespective of their monomeric or dimeric state, which appears contradictory with the dependency of XerCD/*dif* recombination on RecA [59]. In addition, the presence of KOPS all around the chromosome and the fact that FtsK_C_ detached from the septum shows no regional preference suggest that the regional preference is not a property of FtsK by itself. We thus favour the hypothesis that the preferred interaction of FtsK with the FtsK high activity region is a consequence rather than the cause of the delayed segregation. An obvious candidate is the activity of the MatP protein. Although its role is poorly understood, MatP binds specific sites, *matS*, which are found only in the *ter* region and delay its segregation [60]. MatP may thus act to create a proper substrate for FtsK activity into the FtsK high activity region. However, the region containing *matS* sites (from 1135 to 1914) is longer than this region. Another candidate is the persistence of intercatenation links (catenanes) between sister chromosomes. The fact that FtsK appears to be involved in the removal of catenanes argues in favour of this hypothesis. FtsK controls the activity of TopoIV in vitro [21], [22]. Consistent with this observation, TopoIV acts preferentially in the vicinity of the *dif* site [61] and at a late stage of the cell cycle [62]. In addition, in the absence of active TopoIV, XerCD/*dif* recombination can remove catenanes provided FtsK promotes oriented translocation, suggesting that FtsK can control the location of catenation links [63]. Taken together, these data suggest that the removal of catenanes between sister *ter* regions is concomitant with FtsK activity. It follows that catenanes may form or persist preferentially between sister FtsK high activity region and so delay segregation. Clearly, the interplay between FtsK, MatP and TopoIV activities needs to be unravelled if we are to understand the segregation of the *ter* region and its integration in the cell cycle.

## Materials and Methods

### Strains and plasmids

Strains are derived from *E. coli* K12 strain LN2666 (W1485 F^-^ *leu thyA thi deoB or C supE rpsL* (StR)) [64]. The *dif-lacI-dif* [33] and *parS*-Kn cassette [48] were cloned into the *Eco*RV site of plasmid pFC68 [36] to yield plasmid pCP26 and inserted into chromosome-borne Tn*10* or Tc fragments as previously described [36]. The Δ(1364–1589)::Tc, Δ(1387–1391)::psrA*-Ap [39]; Δ(*ftsK_LC_*)-Kn, *ftsK_ATP_*-Cm [15], Δ(*ftsKγ*)-Cm, *ftsK_KOPSblind_*-Cm [31], and Δ(*xerC*)::Gm [52] mutations were transferred by P1 transduction. XerC was produced from plasmid pFC241 (pGB2-*araBADp-xerC*) [33]. FtsK_C_ was produced from plasmid pFX150 (pBAD24-*ftsK_50C_*, [19]). The GFP-Δ30ParB protein was produced from plasmid pALA2705 [56].

### XerCD/*dif* recombination assay

Strains carrying the Δ(*dif*)_58_ [65] and *xerC::*Gm mutations, an insertion of the *dif-lacI-dif* cassette and eventually plasmid pFX150 (FtsK_C_) were grown in L-broth or L-broth plus ampicilin and 0.03% arabinose, rendered competent, and transformed with pFC241 (XerC). Transformants were plated on LB-agar containing 20 µg/ml spectinomycin plus 100 µg/ml ampicilin and 0,025% arabinose when required (pFX150) and grown overnight at 37°C. Five independent transformants were inoculated in the same medium, grown for 5 hours, diluted and plated on L broth plus X-gal (40 µg/ml). The whole procedure corresponds to 20 generations before plating on L-Xgal. The ratio of dark blue to total colonies was used to calculate the frequency of *lacI* loss per generation [37]. The mean of the frequencies of *lacI* loss per generation and standard deviation of 5 independent measures is plotted in the figures.

### Microscopy

Strains carrying a *parS*-Kn insertion and plasmid pALA2705 were grown in M9 medium (0.2% casamino acids, 0.2% glucose; 2 µg/ml thiamine; 20 µg/ml leucine; 20 µg/ml thymine and 100 µg/ml ampicilin) at 30°C to OD_600_ = 0,3 (doubling time ∼90 min.). No inducer (IPTG) was added to keep GFP-Δ30ParB production as low as possible. Cells were plated on poly-L lysine slides, and observed using a Leica DMR-B microscope and a Roper CoolsnapES camera. Images were recorded and processed using Metamorph or ImageJ software.

## Supporting Information

Figure S1 Micrographs of cells with GFP-Δ30ParB/*parS* foci. Strains carrying plasmid pALA2705 and *parS* insertions at the indicated locus were grown and micrographed as indicated in Materials and Methods.
(PDF)
