# Supplementary material for: A Defined Terminal Region of the E. coli Chromosome Shows Late Segregation and High FtsK Activity
Source: PLoS One. 2011 Jul 20;6(7):e22164. doi: 10.1371/journal.pone.0022164 (PMC3140498; doi:10.1371/journal.pone.0022164)
Supplement: Figure S1 — Micrographs of cells with GFP-Δ30ParB/parS foci. Strains carrying plasmid pALA2705 and parS insertions at the indicated locus were grown and micrographed as indicated in Materials and Methods. (PDF) [file pone.0022164.s001.pdf]

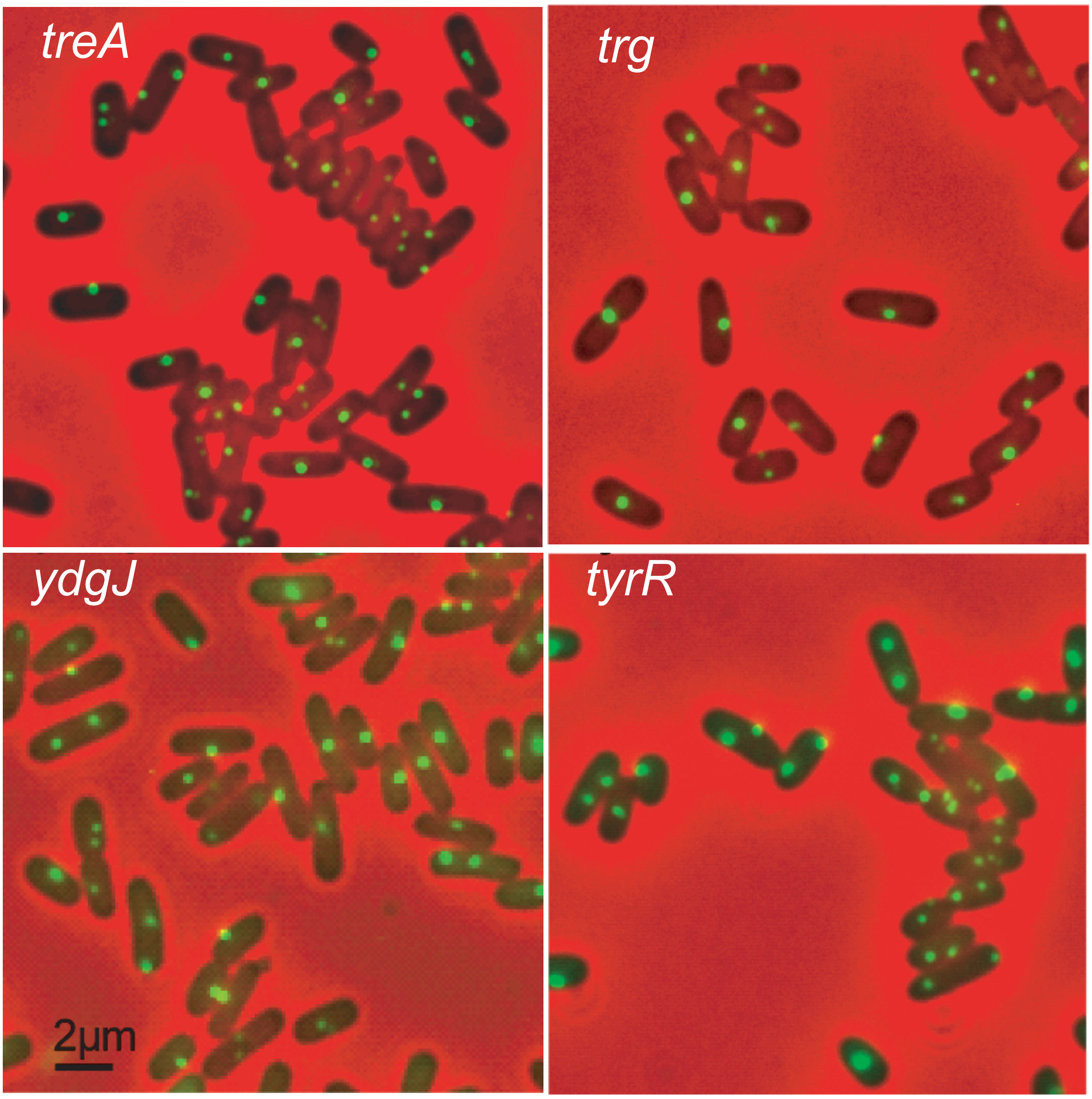

Figure S1: Micrographs of cells with ParB-GFP/*parS* foci.
Strains carrying plasmid pALA2705 and *parS* insertions at the indicated locus were grown and micrographed as indicated in Materials and Methods.
